# A review of evidence supporting amyloid beta reduction as a surrogate endpoint in Alzheimer’s disease

**DOI:** 10.1016/j.tjpad.2025.100458

**Published:** 2026-01-01

**Authors:** Tianle Chen, R. Matthew Hutchison, Carrie Rubel, Jennifer Murphy, Jing Xie, John O’Gorman, Gersham Dent, Geert Molenberghs, Maria Pia Sormani, Suzanne Hendrix, Oskar Hansson, Paul Aisen, Samantha Budd Haeberlein, Ying Tian

**Affiliations:** a Biogen, Inc, 225 Binney Street, Cambridge, MA, 02142, USA; b Hasselt University, Martelarenlaan 42, 3500 Hasselt, Belgium; c Katholieke Universiteit Leuven, Oude Markt 13, 3000 Leuven, Belgium; d University of Genoa, Via Balbi, 5, 16126 Genoa, Italy; e Pentara Corporation, 2261 E 3300 S, Millcreek, UT, 84109, USA; f Clinical Memory Research Unit, Department of Clinical Sciences, Lund University, Box 117, SE-221 00 Lund, Sweden; g Alzheimer’s Therapeutic Research Institute, Keck School of Medicine of University of Southern California, 9860 Mesa Rim Rd, San Diego, CA, 92121, USA

**Keywords:** Surrogate endpoint, Biomarker, Alzheimer’s disease, Amyloid β, Positron emission tomography

## Abstract

Alzheimer’s disease (AD) is a heterogeneous neurodegenerative disease driven by pathological depositions of proteins that accumulate over decades. Compelling genetic and neurobiological evidence suggests that amyloid accumulation in the brain initiates and drives early-stage AD. Measurement of fibrillar amyloid has been pivotal to the development and approval of disease-slowing treatments. Various biomarkers of AD pathophysiology provide evidence of target engagement and downstream effects on disease progression, and their use as surrogate endpoints may help identify and expeditiously bring new treatments to patients. In clinical trials, a surrogate endpoint serves as a substitute for a direct measurement of a patient’s clinical status, and its use can provide ethical, logistical, and economic advantages. Establishing biomarkers as surrogate endpoints involves evaluating scientific evidence through diverse statistical approaches to demonstrate their predictivity of clinical benefit. This article evaluated evidence supporting amyloid β plaque reduction as a surrogate endpoint in symptomatic AD by exploring regulatory considerations and guidelines for surrogate endpoints, examining the amyloid hypothesis and the current therapeutic landscape in AD, and presenting supporting evidence of surrogate endpoints from a recent clinical development program of AD.

## 1 Introduction

This article examines the considerations and methods for the establishment of surrogate endpoints in drug development, with a focus on amyloid beta (Aβ) reduction as a surrogate marker of clinical benefit in Alzheimer’s disease (AD). In Section 2, we review the current regulatory considerations and guidance regarding surrogate endpoints. In Section 3, we examine the amyloid hypothesis, the view that AD is an amyloid-driven tauopathy, and the measurement of amyloid. This is followed by a discussion of the unique complexities that AD interventional trials face when measuring treatment effects. In Section 4, we review the current landscape of anti–amyloid beta (Aβ) monoclonal antibodies as well as the milestones in the development of these therapeutic agents. In Section 5, we present statistical analyses from the recent clinical development programs for AD that provide evidence supporting the reduction in Aβ plaques as a surrogate endpoint for clinical benefit. Finally, in Section 6, we summarize key points and discuss the learnings, limitations, and potential future directions.

## 2 Regulatory considerations for surrogate endpoints

The FDA Center for Drug Evaluation and Research has four expedited programs for the development and review of drugs for serious or life-threatening conditions: Fast Track designation, Breakthrough Therapy designation, Priority Review designation, and Accelerated Approval. In 2023, 36 of 55 (64 %) novel drugs were approved through one or more of these expedited programs; nine (16 %) were approved under the accelerated approval pathway [1]. To date, the FDA has approved more than 300 drugs through the accelerated approval pathway across multiple indications [2]. Historically, in 1992, the FDA began developing a regulatory pathway that allowed for the accelerated approval of drugs for serious conditions with an unmet medical need using a surrogate endpoint [3]. The FDA defines a surrogate endpoint as a marker or other measure that is not itself a direct measure of clinical benefit but rather is known to predict clinical benefit (used to support full approval) or is reasonably likely to predict clinical benefit (used to support accelerated approval) [3,4]. Fully validated surrogate endpoints have a clear mechanistic rationale, have undergone extensive testing, and are supported by robust clinical data [4].

The use of surrogate endpoints can offer important ethical, logistical, and/or economic advantages to speed the development of new treatments, thereby warranting consideration during the planning and preparation stages of a clinical development program. Specifically, by quantifying outcomes that appear earlier or more frequently than a traditional clinical measure, surrogate endpoints can reduce the time patients spend on ineffective treatments and minimize prolonged placebo exposure when waiting for long-term outcomes. As such, utilizing surrogate endpoints can shorten clinical trials, with fewer participants, and deliver faster readouts that lower costs and accelerate development. Since the 1992 FDA guidance on surrogate endpoint use for accelerated approval, surrogate endpoints have been widely adopted for oncology clinical trials. In 2003, the first FDA acceptance was issued based on a reasonably likely surrogate endpoint for bortezomib, a treatment for relapsed or refractory multiple myeloma [5]. Nevertheless, the utility of surrogate endpoints was marred by early setbacks. The FDA-approved drugs encainide, flecainide, moricizine, and zidovudine had been based on candidate surrogate endpoints; however, later postmarketing trials revealed that these surrogate endpoints were poor predictors of clinically relevant outcomes [6]. It had been assumed that there was a surrogacy relationship between a potential surrogate endpoint and the corresponding clinical endpoint. Current regulatory standards now require robust evidence that the surrogate endpoint reliably predicts the effect of treatment for the clinical endpoint that is being replaced by the surrogate [6].

The European Medicines Agency (EMA) has designated a conditional marketing authorization pathway for drugs intended to treat, prevent, or diagnose seriously debilitating or life-threatening disease. The agency may grant conditional marketing authorization if the benefit-risk balance of the medicine is positive, if it is likely that the applicant can provide comprehensive clinical data post authorization, if the medicine fulfills an unmet medical need, and if the benefit of making the medicine available is greater than the inherent risk of requiring additional data. However, the EMA does not specifically mention whether surrogate endpoints may be used to fulfill clinical endpoint requirements [7].

## 3 Role of Aβ in AD and its potential as a surrogate biomarker

### 3.1 Introduction to the aβ hypothesis of AD pathology

AD is a complex, multifactorial, neurodegenerative disease [8,9]. It is characterized by pathophysiological changes in the brain that accumulate decades before clinical symptoms are evident and that continue to evolve after the onset of clinical symptoms [10,11]. The amyloid cascade hypothesis proposes that accumulation of Aβ results from the imbalance between Aβ production and clearance in the brain [12], and the formation of extracellular Aβ plaques is a driving force triggering tau pathology, which is followed by neuronal death [13,14].

The amyloid hypothesis is supported by inheritance patterns observed in autosomal dominant AD and by data from genetic models. Moreover, elevated accumulation of Aβ has been detected in the brains of symptomatic individuals from families carrying autosomal dominant mutations in presenilin 1, presenilin 2, and amyloid beta precursor protein genes compared with the brains of non-carriers [10]. Mutations in the amyloid beta precursor protein gene can result in greater levels of Aβ42, a peptide more prone to fibril formation and the promotion of Aβ aggregates typical of AD [8,[14], [15], [16]]. Sporadic forms of AD are thought to be mechanistically related to the impaired clearance of Aβ [17]. Thus, the upstream accumulation of Aβ has been directly implicated as a causal factor of and proposed therapeutic target for AD [14]. Likewise, soluble oligomers of Aβ, formed by the aggregation of Aβ peptides, have been shown to be neurotoxic and implicated in the pathogenesis of AD [18].

The accumulation of neurofibrillary tangles containing tau have been correlated with cognitive decline and progression in AD [19]. Toxic Aβ species are believed to accelerate the formation of pathological tau by altering the activities of protein kinases and phosphatases that mediate tau phosphorylation and by inducing tau misfolding [19,20]. Thus, a co-dependence exists between Aβ and tau, with Aβ upstream of tau in AD pathogenesis and serving as the trigger for tau conversion. This relationship has been described by the amyloid cascade hypothesis and has become a widely held theory of AD as an amyloid-driven tauopathy [19,20].

Because AD exists on a continuum with variable rates of pathophysiological and clinical progression based on the stage of the disease, biomarker selection is dependent upon the precise population of study. The first stage is amyloid dependent and characterized by amyloid deposits in the basal portions of the isocortex, early changes in amyloid positron emission tomography (PET), and secretion of phosphorylated tau [21,22]. Complexities in patient-level amyloid measurement include substantial individual heterogeneity in genetic drivers of amyloid (e.g. *apolipoprotein E* carrier status), lifestyle factors known to drive baseline amyloid (e.g. diet, exercise, sleep hygiene), individual differences in neuroinflammation function, and co-morbid diseases associated with amyloid accumulation [[23], [24], [25], [26], [27]]. Insoluble tau accumulation is restricted to medial temporal areas in early stages; however, as AD progresses, tau increases in neocortical regions [21], generally following Braak’s proposed temporal/spatial staging hypothesis [22]. The spread of tau to cortical regions is also characterized by increasing density of neurofibrillary tangles and isocortical destruction [22].

### 3.2 Amyloid PET imaging

One of the earliest detectable pathophysiological changes in AD that can be measured by amyloid PET imaging is the accumulation of Aβ plaques. Multiple amyloid radiotracers have been approved for clinical diagnostic use by the FDA: [^18^F]florbetapir (Amyvid; approved in 2013 [28]), [^18^F]flutemetamol (Vizamyl; approved in 2013 [29]), and [^18^F]florbetaben (Neuraceq; approved in 2014 [30]). These radiotracers have also been approved by the EMA for routine clinical diagnostic use and have local regulatory approval in other countries such as Japan and Korea [31]. Premortem amyloid PET autopsy studies performed in end-of-life populations using [^18^F]-labeled radiotracers have shown high sensitivity (88 %−98 %) and specificity (80 %−95 %) for detecting moderate to frequent Aβ plaques at autopsy [[28], [29], [30],^32^]. The tracers do not detect soluble oligomers as these lack the highly ordered β-sheet structure that the PET tracers were designed to target.

Contemporary AD clinical trials have used amyloid PET as a diagnostic biomarker to ensure enrollment of patients with AD who have evidence of brain amyloid pathology. This confirmation can be qualitative (via central reads by a neuroradiologist) or quantitative. Standardization of data collection and image processing enables the quantitative assessment of amyloid burden, in standardized uptake value ratio (SUVR) or centiloid units. It is this quantitation that also allows for longitudinal tracking of changes, which are critical for the use of amyloid PET as a pharmacodynamic biomarker to indicate the biological activity of an investigational therapeutic agent toward Aβ plaques, assist in dose selection, and assess the impact on disease progression [31,[33], [34], [35]].

### 3.3 Challenges of AD interventional trials

Historically, interventional clinical trials conducted in participants with moderate-stage AD used primary endpoints rooted in the regulatory requirement to measure a clinically meaningful, patient-centered experience that globally captured the key disease features of functional decline and cognitive impairment. Dual primary endpoints that separately captured functional and cognitive decline were acceptable, followed by acceptance of using a clinician-administered global interview that captured both (e.g. the Clinical Dementia Rating–Sum of Boxes [CDR-SB]). The FDA’s most recent guidance [2] on the selection of primary endpoints for patients with earlier stage 2 and 3 disease endorses the use of sensitive neuropsychological tests, acknowledging that by definition, patients at this stage do not have functional deficits to measure. Despite these advances in primary endpoint selection, trials remain burdened by unique challenges that make detecting treatment effects difficult. These include (1) the disconnect between pathology and symptomatology (the slow, decades-long buildup of amyloid occurs early in the disease course, then plateaus, years prior to the later-stage emergence of cognitive and functional deficits) [36]; (2) efforts to conduct trials in patients at an earlier disease stage, which are hampered by an inability to detect clinical outcomes because clinical symptoms do not evolve for years, making a trial exceptionally long and impracticable; (3) multiple sources of variability that impact clinical trajectory [37,38], including, for example, individual differences in cognitive function and co-pathologies [39,40]; (4) primary endpoints of clinical outcomes that rely upon the assessment of clinical decline and can be complex and potentially influenced by the rater’s subjectivity and caregiver’s input; and (5) clinical outcome assessments that lack sensitivity and at different stages of the disease can have a restricted range of utility due to both floor and ceiling effects. Given these challenges, clinical trials in AD may benefit from a surrogate biomarker that is able to predict clinical benefit, especially across the earliest symptomatic stages of disease.

Like biomarkers, cognitive and functional outcome measures differ in their appropriateness for disease stage. The CDR-SB is the most commonly used primary outcome measure of global function and is well suited for moderate-stage disease in which the outcome captures both cognitive and functional deficits. At disease stages 2 and 3, this global measure loses sensitivity because these patients have minimal to no functional deficits to capture. Selection of secondary endpoints to measure cognition requires choosing the appropriate version of the cognitive assay (e.g. Alzheimer’s Disease Assessment Scale–Cognitive Subscale [11, 13, or 14 items], and cognitive tests are differentially sensitive to these changes in progression [41,42]. Selection of secondary endpoints to measure function is also chosen based on disease stage (e.g. per the Alzheimer’s Disease Cooperative Study–Instrumental Activities of Daily Living or the Alzheimer’s Disease Cooperative Study–Activities of Daily Living for Mild Cognitive Impairment) because the type of function loss and trajectory of decline differ by disease stage. Development of research frameworks that aid in selecting stage-specific clinical outcome measures are ongoing and will hopefully contribute to the consensus regarding assessment of clinical efficacy.

In 2024, the FDA moved further away from requiring global functional impairment as the basis for approval in the earliest disease stages of AD. They proposed a novel primary endpoint strategy that shifted away from clinical outcome measures [43] and toward biomarkers for the earliest disease stage (stage 1) and biomarkers plus cognition for stage 2. In the modern era of treating patients in earlier disease stages, using biomarkers alone or in combination with cognitive measures are now considered potential primary endpoints, pending discussion with the agency [43].

## 4 Landscape and milestones for anti-Aβ monoclonal antibodies

Several monoclonal antibodies engineered to bind and clear Aβ have advanced to clinical trials and regulatory approvals. First-generation antibodies (e.g. bapineuzumab [Eli Lilly], solanezumab [Eli Lilly], and crenezumab [Genentech/Roche]) had limited clinical activity in patients with prodromal to moderate stages of AD, and many clinical trials of these agents were terminated early [44]. At their tested doses, solanezumab, crenezumab, and bapineuzumab all showed minimal changes in Aβ plaque levels and were not superior to placebo in slowing AD [45].

Clinical trials investigating the efficacy and safety of second-generation anti-Aβ antibodies in patients with early stages of AD (aducanumab [Biogen/Neurimmune], lecanemab [Eisai/Biogen], gantenerumab [Genentech/Roche], and donanemab [Eli Lilly]) required Aβ plaque positivity as one of the inclusion criteria and have all demonstrated a robust effect on reducing brain Aβ plaques as measured by amyloid PET imaging [44,[46], [47], [48], [49]]. Since amyloid PET imaging allows for greater power to measure changes after intervention than clinical assessments, amyloid PET has become a cornerstone, proof-of-concept assessment for current and future anti-Aβ antibodies [50].

**Fig. 1:**
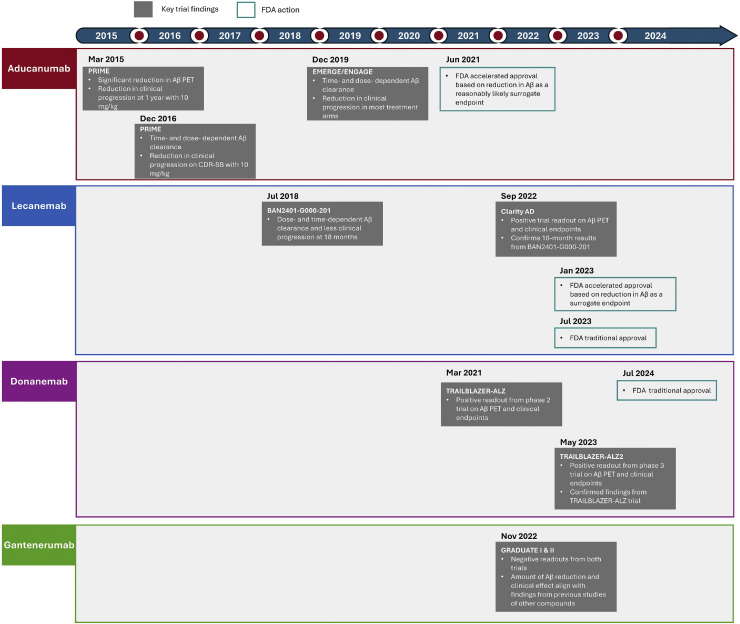
Second-generation anti-Aβ antibodies (aducanumab [60,51,53], lecanemab [[55], [61], [66]], donanemab [[58], [67], [69]], and gantenerumab [64]) are presented. Details on major milestones, FDA approvals, and support for Aβ as a surrogate endpoint. NOTE: Reporting of key clinical trial readouts for each agent is provided in gray boxes. FDA actions are shown in cyan boxes. Aβ, amyloid beta; CDR-SB, Clinical Dementia Rating–Sum of Boxes; FDA, US Food and Drug Administration; MMSE, Mini Mental State Examination; PET, positron emission tomography.

Clinical studies of second-generation anti-Aβ antibodies in which amyloid PET and CDR-SB data were collected are summarized in Supplementary Table 1. The reduction in amyloid plaques observed through PET in several key trials has clarified the relationship between brain amyloid plaque reduction and clinical efficacy and has supported the accelerated FDA approval of aducanumab and lecanemab (Table 1, Fig. 1).

**Table 1:** Milestones for establishing reduction in Aβ plaques as a surrogate endpoint in AD.

**Timing**	**Compound**	**Milestones relevant to surrogate**	**Implication**
March 2015	Aducanumab [51,52]	Phase 1b PRIME study showed a significant reduction in amyloid PET as well as in clinical progression (i.e. CDR-SB and MMSE) at 1 year in the 10-mg/kg treatment group	First demonstration of robust, dose-dependent amyloid PET reduction accompanied by clinical improvement in AD.
December 2016	Aducanumab [53,54]	PRIME study showed a time- and dose-dependent amyloid clearance and a reduction in clinical progression (i.e. CDR-SB) at 1 year in treatment groups receiving fixed and titrated 10-mg/kg dose	The additional cohort further confirmed earlier findings for fixed dosing in the PRIME study
July 2018	Lecanemab [55,56]	Phase 2b BAN2401-G000–201 trial showed a dose- and time-dependent amyloid clearance and less clinical progression (i.e. ADCOMS) at 18 months	Second anti-Aβ drug observed to have effects on Aβ and clinical endpoints
July 2018	– [4]	FDA issued guidance on drug development in early AD	Fully validated surrogate endpoints should have a clear mechanistic rationale, should have undergone extensive testing, and should be supported by robust clinical data
March 2021	Donanemab [57,58]	Positive readout from phase 2 TRAILBLAZER-ALZ trial on amyloid PET and clinical endpoints (i.e. iADRS)	Third anti-Aβ drug observed to have effects on amyloid PET and clinical endpoints
June 2021	Aducanumab [59,60]	FDA issued accelerated approval based on reduction in Aβ plaques (as measured by PET) as a reasonably likely surrogate endpoint	First surrogate endpoint recognized in AD likely to predict clinical benefit
September 2022	Lecanemab [61,62]	Positive readout from phase 3 CLARITY AD trial on amyloid PET and clinical endpoints (i.e. CDR-SB, ADAS-Cog14, ADCOMS, ADCS-MCI-ADL)	Confirmed results of phase 2 Study 201
November 2022	Gantenerumab [63,64]	Negative readouts from phase 3 GRADUATE I and II trials	The amount of Aβ reduction and clinical effect are in line with findings from previous studies of other compounds, supporting Aβ plaque reduction as a surrogate endpoint
January 2023	Lecanemab [65,66]	Phase 2 Study 201, FDA issued accelerated approval based on reduction in Aβ plaques (as measured by PET) as a surrogate endpoint	Second accelerated approval based on this surrogate endpoint in AD
May 2023	Donanemab [67,68]	Positive readout from phase 3 TRAILBLAZER-ALZ 2 trial on amyloid PET and clinical endpoints (i.e. iADRS, CDR-SB, ADAS-Cog13, and ADCS-iADL)	Confirmed its phase 2 findings
July 2023	Lecanemab [66]	FDA issued traditional (full) approval based on phase 3 CLARITY AD trial results, which confirmed the phase 2 findings of reduction in amyloid and clinical outcomes (i.e. ADCOMS, ADAS-Cog14, and CDR-SB)	Confirmed its phase 2 findings. First anti-Aβ compound to receive traditional approval
March 2024	[43]	FDA issued updated draft guidance on drug development in early AD	New content on surrogate endpoint added
July 2024	Donanemab [69]	FDA issued traditional (full) approval based on confirmatory phase 3 TRAILBLAZER-ALZ 2 trial results	Second anti-Aβ compound to receive traditional approval

Abbreviations: Aβ, amyloid beta; AD, Alzheimer’s disease; ADAS-Cog, Alzheimer’s Disease Assessment Scale–Cognitive Subscale; ADCOMS, Alzheimer’s Disease Composite Score; ADCS-iADL, Alzheimer’s Disease Cooperative Study–Instrumental Activities of Daily Living; ADCS-MCI-ADL, Alzheimer’s Disease Cooperative Study–Activities of Daily Living for Mild Cognitive Impairment; CDR-SB, Clinical Dementia Rating–Sum of Boxes; FDA, US Food and Drug Administration; iADRS, Integrated Alzheimer’s Disease Rating Scale; MMSE, Mini Mental State Examination; PET, positron emission tomography.

## 5 Statistical analyses supporting reduction in Aβ as a surrogate endpoint in AD

Various statistical approaches for establishing surrogate endpoints have been proposed over the past decades, including the Prentice criteria [70], the proportion of treatment effect explained [71], and the causal inference frameworks [72,73]. These approaches, which analyze data from a single trial, are stringent and may only work well in the ideal setting of surrogate endpoints [74]. The meta-analytic approach, proposed by Buyse et al., assesses the individual-level and treatment group–level correlation simultaneously using individual-level data from multiple trials [75]. General meta-analyses that directly use the treatment group–level estimates from publicly available information are more widely used given the data accessibility. For a detailed review of the methodology and corresponding real-world applications, see Chen et al. [74]. In this section, we review four statistical approaches that are most relevant to support the surrogacy of reduction in Aβ plaques in AD.

### 5.1 Treatment group–level correlation analysis

Treatment group–level correlation assesses the relationship between the control-adjusted treatment effects on the biomarker and on the clinical endpoint from each of the active treatment groups across multiple studies and/or therapeutic agents. It leverages the fundamental advantages of randomized controlled trials in that (1) it directly assesses the association between the control-adjusted treatment benefits on biomarkers and on clinical endpoints, which directly aligns with the requirement for a surrogate endpoint to predict clinical benefit and (2) participant heterogeneity is addressed by randomization, and outcome heterogeneity (clinical and possibly biomarker) is addressed by using adjusted group mean-level estimates [76]. Given the definition of clinical benefit (the difference between treatment group and control group in the main clinical outcome), treatment group–level correlation is a necessary condition for establishing surrogacy, even if it is not sufficient, being an ecological correlation. Biological plausibility is a key requirement for establishing surrogacy in this context. This approach has been widely used for establishing surrogate endpoints in multiple disease areas, including oncology, HIV, and cardiovascular disease [6,77].

Treatment group–level correlation has recently been possible in AD because of the use of the harmonized centiloid scale for amyloid PET and the emergence of recent data from multiple clinical trials in early symptomatic AD of anti-Aβ antibodies with a similar mechanism of action [78]. A centiloid value of zero represents the population mean level of Aβ-negative individuals, while a value of 100 represents the population mean level of Aβ burden in patients with of mild to moderate dementia severity due to AD [78]. Aducanumab and lecanemab had a wide range of dose levels in their proof-of-concept studies, which makes it possible to conduct the treatment group–level analysis with the existing data. However, the single dose level of donanemab in phase 2 and 3 studies prevented the treatment group–level analysis from being conducted.

Treatment group–level correlation analyses were conducted in the aducanumab trials for the PRIME, EMERGE, and low-dose ENGAGE cohorts. When the adjusted mean difference from placebo in the CDR-SB was plotted against the adjusted mean difference in amyloid PET composite SUVR, a greater treatment effect on brain Aβ plaque levels was associated with a greater clinical benefit [59]. This analysis was done using the SUVR scale since only one amyloid PET tracer, [^18^F]florbetapir, was used for longitudinal amyloid PET assessment in the phase 1b and 3 trials.

A similar analysis was conducted across the five active dose arms in the lecanemab phase 2 trial (Study 201), which showed similar results [65]. Consistent parallel directional relationships between biomarker changes and changes in clinical measures were noted. Specifically, the reduction in brain Aβ with lecanemab treatment as measured by amyloid PET was associated with a slowing of clinical decline as assessed by the change in the CDR-SB (Pearson correlation [*r*]=0.802, *P*=.103; Spearman correlation [ρ]=0.800, *P*=.104) [65].

In 2022 and 2023, data from additional phase 3 clinical studies were reported for lecanemab (CLARITY AD), gantenerumab (GRADUATE I and GRADUATE II), and donanemab (TRAILBLAZER-ALZ 2). In an exploratory analysis, Chen et al. reported treatment group–level correlations between the adjusted mean difference from placebo in the CDR-SB and the adjusted mean difference from placebo in amyloid PET using clinical trial data for second-generation anti-Aβ antibodies from up to 2023. The sample size–weighted Spearman correlation including all data points and excluding the ENGAGE high-dose treatment group (see Section 5.1 for details) was 0.78 and 0.84, respectively [76]. Here, the rank-based Spearman correlation can be applied to any monotone relationship without the assumption on linearity, which makes the approach more general and robust to outliers.

A negative-control simulation analysis was conducted to quantify the probability of false-positive correlation by shuffling and randomly drawing pairs of the treatment effect on amyloid PET and clinical benefit from the results of the observed studies. The probability of observing a false-positive correlation coefficient larger than 0.7 is extremely low (≈0.05 %) [79]. The evolution of the treatment group–level correlation analysis in AD suggested that with the cumulative supporting data from the emerging studies, the probability of false positivity becomes lower and consequently, the confidence in the surrogate endpoint becomes higher in the field.

Comparatively, a similar analysis was conducted by Zhu et al., which included studies of the first-generation anti-Aβ antibodies bapineuzumab, solanezumab, crenezumab, and gantenerumab [45]. The gantenerumab data used in their analysis came from the SCarlet RoAD trial (105 or 225 mg every 4 weeks), whereas Chen et al. included data from the GRADUATE I and II trials (target dose of 1020 mg every 4 weeks) [76]. At the doses analyzed by Zhu et al., solanezumab, crenezumab, bapineuzumab, and gantenerumab all showed minimal changes in Aβ plaque levels as assessed by PET SUVR and in clinical benefit per the CDR-SB, thereby lacking superiority over placebo in slowing AD [45]. The magnitude of SUVR reduction for all past first-generation anti-Aβ antibody trials was quite small (≤0.1 unit) compared with the reduction seen with aducanumab, lecanemab, and donanemab, indicating that the former trials failed to achieve a meaningful clinical endpoint change [80].

More recently, Wang et al. confirmed that the relationship between changes in amyloid PET and changes in the CDR-SB was relevant by including data for lanabecestat, semagacestat, and verubecestat, all of which had negative findings in large phase 3 trials. As in prior trials, the magnitude of SUVR reduction was not large enough to achieve a meaningful change in the clinical endpoint, and thus served as a useful negative control [80]. The inclusion of additional trial data in these analyses increased their statistical rigor, while the inclusion of both positive and negative data increased the accuracy and precision of trial-level surrogacy assessment [6,81].

Among the aducanumab, lecanemab, and donanemab studies, clear reductions in Aβ were seen, as assessed with PET. The high-dose aducanumab group from the phase 3 ENGAGE trial was an outlier. In this study, high-dose aducanumab treatment showed negative efficacy on the CDR-SB, unlike low-dose aducanumab [45]. Considerable work was conducted to explain these discordant results. Post hoc analyses revealed that outcomes in the aducanumab high-dose group in ENGAGE were affected by an imbalance in a small number of patients with extremely rapid progression and by lower exposure to the target 10-mg/kg dose [82]. When the incidence of rapidly progressing patients was balanced across treatment arms in ENGAGE and EMERGE, the results were found to be consistent across studies in later-enrolled patients.

### 5.2 Treatment group–level threshold analysis

In addition to the linear correlation analysis, a treatment group–level threshold approach has been proposed in recent years. In this approach, the residual of the amyloid PET burden after treatment is correlated with the clinical benefit in a binary way: the treatment groups with amyloid PET residual below the positivity threshold show a positive clinical benefit (specifically meaning a statistically significant result on the primary clinical endpoint), while the treatment groups with amyloid PET residual above the threshold do not show a clinical benefit.

Although a threshold for effect on amyloid PET burden has not been widely agreed upon, when statistically significant clinical outcomes have been observed, this threshold usually falls within the range of 20 to 30 centiloids [31,83]. A reduction below 30 centiloids is conveniently close to the cutoff for PET visual reads assessed at baseline to include individuals in the trial (i.e. the reduction threshold and the baseline threshold for amyloid positivity are similar), although no inference can be drawn from this result.

The threshold approach investigates the treatment group–level relationship between amyloid PET and clinical endpoints from a different perspective than the usual linear/monotone relationship. However, a limitation of this approach is that it is based on a binary transform of the *P* value for the effect on the clinical endpoint, which is neither a clinically useful nor a quantitative value. Whether a trial is considered positive or negative may be due, at least in part, to the sample size. For example, when taken separately, gantenerumab treatment in the GRADUATE I and GRADUATE II trials failed to produce a significant clinical effect on the CDR-SB [63,84]. However, when the GRADUATE I and II trial data were pooled, the effect of gantenerumab became statistically significant on the CDR-SB, with an intermediate amyloid reduction [63]. Effects on both amyloid reduction and cognition are clearly shown by the linear correlation analysis of continuous variables. Therefore, this treatment group–level threshold analysis is not recommended due to the aforementioned limitation of statistical meaningfulness. Beyond this conceptual limitation, the results obtained using this approach do not conflict with the linear approach in that the residual is calculated as the baseline amyloid level minus total amyloid removed.

### 5.3 Individual-level correlation analysis

Amyloid PET is a biomarker of an early and accelerating event (i.e. a biomarker that captures early pathological changes prior to symptom manifestation, with minimal progression expected during the typical early AD clinical trial window). Therefore, a correlation between an individual’s change from baseline in amyloid PET and change from baseline in clinical endpoints can only happen when a treatment is effective in removing Aβ plaques and slowing down clinical progression simultaneously [76].

In the placebo arm, no correlation is expected due to the stable Aβ level at this stage of the disease. In the positive aducanumab EMERGE study, a statistically significant correlation in the hypothesized direction was observed for all four clinical endpoints at Week 78 (Supplementary Table 2) [59]. Placebo results served as a negative control to support the claim of treatment-induced correlation in the active treatment groups. Partial Spearman correlation was used to (1) adjust for baseline biomarker and clinical endpoint values and (2) handle outlier data points. The rank-based Spearman correlation does not require the linearity assumption, and thus is robust to non-normal data distributions and outliers. Considering the potential time lag between the biomarker and the clinical endpoint, a similar pattern emerged when the correlation between the change from baseline in amyloid PET at Week 78 and the change from baseline in the clinical endpoints at Week 106 was assessed [85].

Individual-level correlation analyses have been reported for other second-generation anti-Aβ antibody compounds as well [65,86,57]. These analyses differ in the following aspects: (1) different timepoints were used, some due to the different study duration by design, and some due to the sponsor’s choice; (2) some analyses pooled the placebo and active arms, and some separated the placebo participants from the treated participants; (3) different endpoints of amyloid PET were used, including change from baseline, percent change from baseline, and residual value at the timepoint of interest; and (4) some analyses used the Pearson correlation with linearity assumption, and some used the rank-based Spearman correlation. Chen et al. provided recommendations on how to conduct the individual-level correlation for biomarkers in AD [76]. Regardless of these differences in analytical methods, the results indicated a general pattern consistent with a treatment-induced individual-level correlation between amyloid PET and clinical endpoints, albeit modest, in most cases. The magnitude of the individual-level correlation is not surprising given the heterogeneity of the patient population and the large variability in the clinical scales.

As explained by Chen et al., only for early accelerating biomarkers (i.e. those in which changes significantly precede the onset of clinical symptoms [76]) such as amyloid PET, the treatment-induced individual-level correlation may support the association between treatment effect on a biomarker and treatment effect on a clinical endpoint, thus supporting its use as a surrogate biomarker [76]. Judgment needs to be exercised for other biomarkers. For late accelerating biomarkers (i.e. biomarkers that capture pathological changes during the symptom manifestation of the disease), treatment-induced individual-level correlation is confounded by the prognostic association between the natural progression of the two endpoints.

### 5.4 Individual-level threshold analysis

**Fig. 2:**
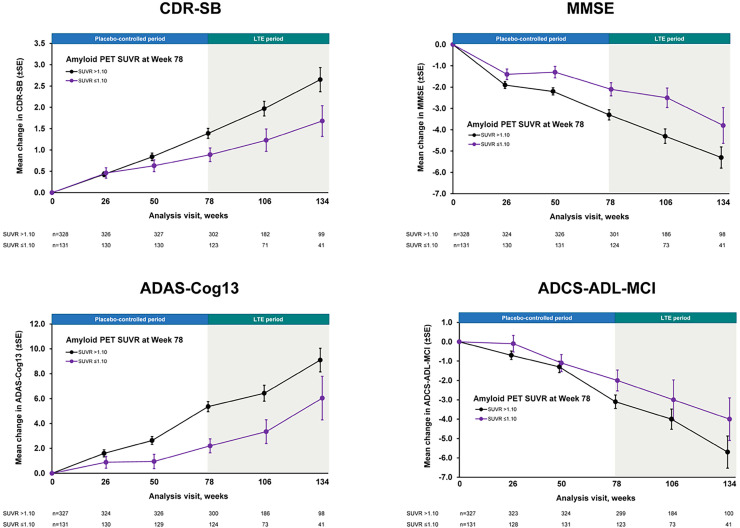
Long-term clinical decline by amyloid PET status in aducanumab EMERGE and ENGAGE studies [87]. NOTE: Clinical outcomes were assessed by amyloid PET SUVR in pooled patients from low- and high-dose aducanumab treatment groups in EMERGE and ENGAGE. Pooled data were collected during the placebo-controlled and LTE periods and subsequently stratified by SUVR threshold (>1.10 vs ≤1.10). A PET SUVR value of 1.10 is equivalent to a centiloid value of 20.2. ADAS-Cog13, Alzheimer’s Disease Assessment Scale–Cognitive Subscale (13 items); ADCS-ADL-MCI, Alzheimer’s Disease Cooperative Study–Activities of Daily Living Inventory for Mild Cognitive Impairment; CDR-SB, Clinical Dementia Rating–Sum of Boxes; LTE, long-term extension; MMSE, Mini Mental State Examination; PET, positron emission tomography; SE, standard error; SUVR, standardized uptake value ratio.

In addition to the linear relationship, the clinical benefit in subgroups of individuals who meet the threshold versus those who do not was assessed using a categorical threshold method to examine individual-level data. On average, patients who reached this threshold after receiving treatment showed a slower clinical decline than those who did not (Fig. 2) [87]. A smaller magnitude of decline was observed in the placebo-controlled period and a continued trend was observed in the long-term extension, providing evidence of an association between treatment-induced Aβ reduction and clinical benefit at a categorical level. It should be noted that a [^18^F]florbetapir SUVR value of 1.10 is equivalent to a centiloid value of 20.2 [88]. A similar amyloid-positivity threshold analysis was conducted using gantenerumab data from the GRADUATE I and II studies [89]. Patients treated with gantenerumab whose amyloid values fell below the threshold showed a slower clinical decline, consistent with the observations in EMERGE and ENGAGE. However, the authors suggested that potential confounding factors in baseline characteristic imbalances (older age, lower body weight, earlier in disease course) and a small sample size may have accounted for these observations [89].

The individual-level threshold analysis is subject to potential bias due to (1) stratification of patients based on a post-baseline factor and (2) exclusion of patients with missing data on the timepoint used for the threshold. Therefore, it should be interpreted with caution.

In summary, these four statistical approaches conducted on seven anti-Aβ antibody compounds across more than a dozen phase 1 to 3 studies provide consistent and substantial evidence supporting the reduction of Aβ plaques in AD as a surrogate endpoint. Table 2 summarizes the strengths and limitations of each approach. Some, like the treatment group–level correlation analysis, are more applicable to the surrogacy framework and are more meaningful. Other approaches may be better suited to sensitivity analyses, with their limitations noted. It is important to clearly understand each statistical approach in evaluating surrogate endpoints and apply them appropriately in each situation.

**Table 2:** Strengths and limitations of statistical approaches.

**Approach**	**Key features**	**Strengths**	**Limitations**
Treatment group–level correlation analysis	Assesses the relationship between treatment effects on Aβ and clinical outcomes across trials/doses; uses control-adjusted treatment differences	Leverages randomization; directly assesses predictive value; robust, with multiple trial results; less affected by patient heterogeneity	Requires multiple trials/doses; needs harmonized measurements
Treatment group–level threshold analysis	Binary categorization of amyloid based on treatment group–level residual amyloid levels; evaluates clinical outcomes by threshold achievement (*P*<.05)	Simple interpretation	Loss of information from dichotomization; arbitrary threshold on clinical outcomes that depends on sample size
Individual-level correlation analysis	Examines within-patient association between Aβ changes and clinical outcomes; can adjust for baseline values	Uses all individual patient data; can assess within-trial relationships; enables covariate adjustment	Affected by patient heterogeneity; may not reflect treatment effects; sensitive to missing data
Individual-level threshold analysis	Binary categorization of amyloid based on individual-level residual amyloid levels; evaluates clinical trajectory in each category	Simple interpretation; clinically meaningful cutoffs for amyloid PET	Loss of information from dichotomization; stratify patients based on a post-baseline factor; sensitive to missing data

For additional information on individual-level and treatment-group level methodology and real-world applications please see the publications by Chen et al. [74] and Buyse et al. [75].

Abbreviations: Aβ, amyloid beta; PET, positron emission tomography.

## 6 Discussion

A surrogate endpoint serves as a substitute for directly measuring a patient’s clinical status and progression. It offers several economic and safety advantages for clinical trial design while speeding up the approval of promising new therapies for unmet medical needs compared with traditional pathways [[3], [6], [81], [90], [91]]. Surrogate endpoints have been used in medical research for several decades, marked by both failures and successes [6]. While this trend is most evident in the field of oncology, the use of biomarkers as surrogate endpoints has now entered the field of AD as well [11,43,[60], [92], [93]].

The clinical validity of a surrogate endpoint is based on the biological plausibility of the disease pathway and its ability to predict clinical benefit. Clinical benefit is the treatment effect on the clinical efficacy endpoint, defined as the difference in treatment response between the active treatment group and the control group. Therefore, (1) clinical benefit is a treatment group-level rather than an individual-level quantity and (2) a control (e.g. placebo) group is needed to calculate the control-adjusted treatment effect [76]. Randomized controlled trials are the most appropriate setting to assess clinical benefit and are thus critical for establishing a surrogate endpoint. In pivotal stage 3 and later clinical trials, the outcome measure that defines clinical benefit should detect a clinically meaningful change in how the patient feels, functions, or survives. In stage 2 trials, sensitive cognitive outcomes can qualify as “clinical benefit,” provided that their is alignment with the FDA [43].

The treatment group–level correlation analysis leverages the fundamental advantages of randomized controlled trials and is an appropriate approach to assess the surrogacy of endpoints [76]. However, meta-analyses conducted by different groups may lead to inconsistent findings for the same surrogate endpoint. For example, Ackley et al. concluded that amyloid reduction does not substantially improve cognition based on a variable meta-analysis of 14 randomized controlled trials [94,95]. When repeating the meta-analysis after correcting for identified data inconsistencies and adding new trial data, a statistically significant causal relationship between amyloid removal and cognitive and functional decline was found by Pang et al. [96]. The disease-modifying effects of an anti-Aβ agent might also be delayed, suggesting that the follow-up time of trials may need to be extended to have sufficient statistical power [97]. Thorough and logical selection of trials, transparent reporting of trial characteristics, and consistent reporting of analysis methods can help highlight the potential issues that arise during the analysis and avoid drawing biased conclusions.

Treatment group–level correlation analysis is recognized as a critical piece of evidence for establishing the surrogacy of Aβ reduction in AD. Consistent results using multiple statistical approaches on the same datasets from multiple studies of different anti-Aβ antibodies are supportive [76,80]. In addition, pharmacological exposure-response analyses further support the relationship between aducanumab treatment and longitudinal responses across aducanumab clinical trials [80]. A deep understanding of disease biology and careful consideration of each case are required to identify which appropriate statistical approaches to use [76].

The causes of variability in amyloid PET, including differences in the amyloid tracer used, method of analysis, target and reference regions used, and use of partial volume correction, demonstrate the need to standardize methods. One such method is the centiloid scale, which harmonizes treatment group–level data across multiple AD clinical trials and further supports the use of meta-analytical evaluations. The harmonization efforts for tau PET are ongoing [98,99].

Modeling treatment effects on multiple surrogate outcomes simultaneously has been proposed as a way to remove some measurement error and reduce prediction uncertainty and has been recommended by the National Institutes of Health Workshop on the use of surrogate endpoints as a goal of future research [90,100]. Research on the validation of multiple surrogate endpoints is currently being explored in HIV and multiple sclerosis [90,100]. Further, Bujkiewicz et al. have proposed Bayesian meta-analytical methods to incorporate multiple surrogate endpoints in the drug development process [90]. Considering the complex biological mechanisms in many diseases and the new biomarkers emerging from the rapid advancements in biological research, efforts to explore a combination of surrogate biomarker profiles is warranted.

To date, the FDA has supported the use of Aβ reduction as a surrogate endpoint in AD clinical trials for accelerated approval [101]; however, whether this surrogate endpoint becomes fully validated and used for traditional approval remains to be seen [102]. In some cases, regulatory agencies may leverage surrogate validation studies that have been conducted using internal or external data [103]. Thus, further trial-level and statistical validation of Aβ as a surrogate endpoint may add to the totality of information supporting future regulatory decisions, including robust data showing that reduction in Aβ that has been shown to be strongly associated with AD clinical outcomes, is tied to mechanistic changes in AD progression, and manifests early during AD. While there is evidence supporting the surrogacy of Aβ reduction in symptomatic AD, further research is required to support its use in preclinical AD. Forthcoming data readouts from the AHEAD 3–45 study may provide insight into surrogacy in preclinical and early preclinical AD [104]. Although amyloid plaques represent a risk factor for neurodegeneration, they may also occur in the absence of substantial cell death. Accordingly, while amyloid reduction has demonstrated value as a surrogate marker, its change is not a prerequisite for demonstrating disease modification in AD. Amyloid PET primarily detects insoluble fibrillar aggregates and lacks sensitivity to soluble oligomeric species. The therapeutic strategies targeting these soluble forms, as well as tau pathology, neuroinflammation, or other pathological processes, may yield minimal change in amyloid PET signal yet confer significant clinical benefit. Further, as emerging data and novel interventions refine our understanding of disease biology, the repertoire of surrogate endpoints in AD is likely to expand, potentially encompassing tau PET, fluid biomarkers, and other modality-specific measures.

## 7 Conclusion

This review discussed the evolving evidence supporting the use of Aβ reduction as a surrogate endpoint for predicting clinical benefit in AD. Multiple statistical approaches, including treatment group–level and individual-level correlations as well as threshold analyses, consistently showed a relationship between amyloid reduction and clinical outcomes across several second-generation anti-Aβ antibody trials. The use of surrogate endpoints has facilitated accelerated approvals and may significantly expedite future drug development in AD. This review underscores the potential importance of surrogate endpoints in AD drug development, while highlighting the need for ongoing research to fully establish their clinical validity.

## Sources of funding

Biogen funded this work. Biogen authors contributed to the analysis and interpretation of data in this review, in the writing of the manuscript; and in the decision to submit this article for publication.

## Declaration of generative AI and AI-assisted technologies in the writing process

AI or AI-assisted technologies were not used in the writing process of this manuscript.

## CRediT authorship contribution statement

**Tianle Chen:** Writing – review & editing, Writing – original draft, Visualization, Project administration, Conceptualization. **R. Matthew Hutchison:** Writing – review & editing, Writing – original draft, Visualization, Project administration, Conceptualization. **Carrie Rubel:** Writing – review & editing. **Jennifer Murphy:** Writing – review & editing. **Jing Xie:** Writing – review & editing. **John O’Gorman:** Writing – review & editing. **Gersham Dent:** Writing – review & editing. **Geert Molenberghs:** Writing – review & editing. **Maria Pia Sormani:** Writing – review & editing. **Suzanne Hendrix:** Writing – review & editing. **Oskar Hansson:** Writing – review & editing. **Paul Aisen:** Writing – review & editing. **Samantha Budd Haeberlein:** Writing – review & editing. **Ying Tian:** Writing – review & editing, Project administration, Conceptualization.

## Declaration of competing interest

The authors declare the following financial interests/personal relationships which may be considered as potential competing interests: R. Matthew Hutchison, Carrie Rubel, Jennifer Murphy, Jing Xie, John O’Gorman, and Gersham Dent are employees and shareholders of Biogen Inc. Tianle Chen, Samantha Budd Haeberlein, and Ying Tian were employees of Biogen at the time of this study and have since left the company. Geert Molenberghs consults with the Critical Path for Alzheimer’s Disease (CPAD) initiative of the Critical Path Institute on the evaluation of tau as a potential surrogate marker in AD. Maria Pia Sormani has received consulting fees from Alexion, Biogen, Immunic, Merck, Novartis, Roche, and Sanofi. Suzanne Hendrix is CEO of Pentara Corporation, has received consultancy/speaker fees from Biogen, and consults with many companies in the AD field. Oskar Hansson has received research support (for the institution) from Avid Radiopharmaceuticals, Biogen, Eli Lilly, Eisai, GE HealthCare, Pfizer, and Roche. In the past 2 years, he has received consultancy/speaker fees from AC Immune, ALZpath, Biogen, Cerveau, and Roche. Paul Aisen is Chair of the steering committee of the aducanumab program at the Alzheimer’s Therapeutic Research Institute, Keck School of Medicine of University of Southern California; has received research support from Eli Lilly, Janssen, Eisai, the Alzheimer’s Association, the National Institutes of Health, and the Foundation for the National Institutes of Health; and has consulted for Merck, Roche, and ImmunoBrain Checkpoint. If there are other authors, they declare that they have no known competing financial interests or personal relationships that could have appeared to influence the work reported in this paper.

## Appendix Supplementary materials

Image, application 1

## References

[1] Advancing health through innovation: new drug therapy approvals 2023. Center for Drug Evaluation and Research. Food and Drug Administration; September 19, 2024.

[2] Drug CDER (2024). Biologic Accelerated Approvals Based on a Surrogate Endpoint. US Food and Drug Administration.

[3] U.S. Food and Drug Administration. Accelerated approval. 2023 https://www.fda.gov/patients/fast-track-breakthrough-therapy-accelerated-approval-priority-review/accelerated-approval. September 19, 2024.

[4] U.S. Food and Drug Administration. Surrogate endpoint resources for drug and biologic development. 2018 https://www.fda.gov/drugs/development-resources/surrogate-endpoint-resources-drug-and-biologic-development. September 18, 2024.

[5] Bross P.F., Kane R., Farrell A.T., Abraham S., Benson K., Brower M.E. (2004). Approval summary for bortezomib for injection in the treatment of multiple myeloma. Clin Cancer Res.

[6] Buyse M., Molenberghs G., Paoletti X., Oba K., Alonso A., Van der Elst W. (2016). Statistical evaluation of surrogate endpoints with examples from cancer clinical trials. Biom J.

[7] European Medicines Agency. Conditional marketing Authorization. 2004 https://www.ema.europa.eu/en/human-regulatory-overview/marketing-authorisation/conditional-marketing-authorisation. September 19, 2024.

[8] Breijyeh Z., Karaman R. (2020). Comprehensive review on Alzheimer's Disease: causes and treatment. Molecules.

[9] Khan S., Barve K.H., Kumar M.S. (2020). Recent advancements in pathogenesis, diagnostics and treatment of Alzheimer's disease. Curr Neuropharmacol.

[10] Monteiro A.R., Barbosa D.J., Remião F., Silva R. (2023). Alzheimer's disease: insights and new prospects in disease pathophysiology, biomarkers and disease-modifying drugs. Biochem Pharmacol.

[11] Porsteinsson A.P., Isaacson R.S., Knox S., Sabbagh M.N., Rubino I. (2021). Diagnosis of early Alzheimer's Disease: clinical practice in 2021. J Prev Alzheimers Dis.

[12] Reynolds D.S. (2019). A short perspective on the long road to effective treatments for Alzheimer's disease. Br J Pharmacol.

[13] Arnsten A.F.T., Datta D., Del Tredici K., Braak H. (2021). Hypothesis: tau pathology is an initiating factor in sporadic Alzheimer's disease. Alzheimers Dement.

[14] Hampel H., Hardy J., Blennow K., Chen C., Perry G., Kim S.H. (2021). The amyloid-β pathway in Alzheimer's disease. Mol Psychiatry.

[15] Tcw J., Goate A.M. (2017). Genetics of β-amyloid precursor protein in Alzheimer's disease. Cold Spring Harb Perspect Med.

[16] Schreiner T.G., Schreiner O.D., Adam M., Popescu B.O (2023). The roles of the amyloid beta monomers in physiological and pathological conditions. Biomedicines.

[17] Li Y., Rusinek H., Butler T., Glodzik L., Pirraglia E., Babich J. (2022). Decreased CSF clearance and increased brain amyloid in Alzheimer's disease. Fluids Barriers CNS.

[18] Viola K.L., Klein W.L. (2015). Amyloid β oligomers in Alzheimer's disease pathogenesis, treatment, and diagnosis. Acta Neuropathol.

[19] Bloom G.S. (2014). Amyloid-β and tau: the trigger and bullet in Alzheimer disease pathogenesis. JAMA Neurol.

[20] Sheng L., Bhalla R. (2024). Biomarkers and target-specific small-molecule drugs in Alzheimer's diagnostic and therapeutic research: from amyloidosis to tauopathy. Neurochem Res.

[21] Jack C.R., Andrews S.J., Beach T.G., Buracchio T., Dunn B., Graf A. (2024). Revised criteria for the diagnosis and staging of Alzheimer's disease. Nat Med.

[22] Braak H., Braak E. (1991). Neuropathological stageing of Alzheimer-related changes. Acta Neuropathol.

[23] Chen G., McKay N.S., Gordon B.A., Liu J., Joseph-Mathurin N., Schindler S.E. (2024). Predicting cognitive decline: which is more useful, baseline amyloid levels or longitudinal change?. NeuroImage: Clin.

[24] Díaz G., Lengele L., Sourdet S., Soriano G., de Souto, Barreto P. (2022). Nutrients and amyloid β status in the brain: a narrative review. Ageing Res Rev.

[25] Brown B.M., Sohrabi H.R., Taddei K., Gardener S.L., Rainey-Smith S.R., Peiffer J.J. (2017). Habitual exercise levels are associated with cerebral amyloid load in presymptomatic autosomal dominant Alzheimer's disease. Alzheimers Dement.

[26] Lucey B.P., Hicks T.J., McLeland J.S., Toedebusch C.D., Boyd J., Elbert D.L. (2018). Effect of sleep on overnight cerebrospinal fluid amyloid β kinetics. Ann Neurol.

[27] Sedaghat S., Ji Y., Hughes T.M., Coresh J., Grams M.E., Folsom A.R. (2023). The association of kidney function with plasma amyloid-β levels and brain amyloid deposition. J Alzheimers Dis.

[28] Amyvid (florbetapir F-18) [prescribing information]. Lilly; Indianapolis, IN. 2023.

[29] Vizamyl (flutemetamaol F-18) [prescribing information]. GE Healthcare; Arlington Heights, IL. 2017.

[30] Neuraceq (florbetaben) [prescribing information]. Life Molecular; Hampshire, UK. 2024.

[31] Pemberton H.G., Collij L.E., Heeman F., Bollack A., Shekari M., Salvadó G. (2022). Quantification of amyloid PET for future clinical use: a state-of-the-art review. Eur J Nucl Med Mol Imaging.

[32] Lesman-Segev O.H., La Joie R., Iaccarino L., Lobach I., Rosen H.J., Seo S.W. (2021). Diagnostic accuracy of amyloid versus (18) F-fluorodeoxyglucose positron emission tomography in autopsy-confirmed dementia. Ann Neurol.

[33] Bader I., Bader I., Lopes Alves I., Vállez García D., Vellas B., Dubois B. (2023). Recruitment of pre-dementia participants: main enrollment barriers in a longitudinal amyloid-PET study. Alzheimers Res Ther.

[34] Hayato S., Takenaka O., Sreerama Reddy S.H., Landry I., Reyderman L., Koyama A. (2022). Population pharmacokinetic-pharmacodynamic analyses of amyloid positron emission tomography and plasma biomarkers for lecanemab in subjects with early Alzheimer's disease. CPT Pharmacomet Syst Pharmacol.

[35] Schuck E.L., Reyderman L., Hayato S., Takenaka O., Swanson C.J., Lai R.Y.K. (2019). Population pharmacokinetic/pharmacodynamic analyses of BAN2401 in patients with early Alzheimer's disease: correlation of BAN2401 exposure, PET standard uptake value ratio, and cognitive outcomes. Alzheimers Dement.

[36] Sperling R., Mormino E., Johnson K. (2014). The evolution of preclinical Alzheimer's disease: implications for prevention trials. Neuron.

[37] Cloutier S., Chertkow H., Kergoat M.J., Gauthier S., Belleville S. (2015). Patterns of cognitive decline prior to dementia in persons with mild cognitive impairment. J Alzheimers Dis.

[38] Cohen C.I., Reisberg B., Yaffee R. (2024). Global cognitive trajectory patterns in Alzheimer's disease. Int Psychogeriatr.

[39] Tosun D., Hausle Z., Iwaki H., Thropp P., Lamoureux J., Lee E.B. (2024). A cross-sectional study of α-synuclein seed amplification assay in Alzheimer's disease neuroimaging initiative: prevalence and associations with Alzheimer's disease biomarkers and cognitive function. Alzheimers Dement.

[40] Van Der Gaag B.L., Deshayes N.A.C., Breve J.J.P., Bol J., Jonker A.J., Hoozemans J.J.M. (2024). Distinct tau and alpha-synuclein molecular signatures in Alzheimer's disease with and without lewy bodies and Parkinson's disease with dementia. Acta Neuropathol.

[41] Maheux E., Koval I., Ortholand J., Birkenbihl C., Archetti D., Bouteloup V. (2023). Forecasting individual progression trajectories in Alzheimer's disease. Nat Commun.

[42] Jutten R.J., Papp K.V., Hendrix S., Ellison N., Langbaum J.B., Donohue M.C. (2023). Why a clinical trial is as good as its outcome measure: a framework for the selection and use of cognitive outcome measures for clinical trials of Alzheimer's disease. Alzheimers Dement.

[43] Early Alzheimer's disease: developing drugs for treatment - guidance for industry. 2024 https://www.fda.gov/media/110903/download. September 17, 2024.

[44] Tian Hui Kwan A., Arfaie S., Therriault J., Rosa-Neto P., Gauthier S. (2020). Lessons learnt from the second generation of anti-amyloid monoclonal antibodies clinical trials. Dement Geriatr Cogn Disord.

[45] Zhu H., Mehta M., Huang S.M., Wang Y. (2022). Toward bridging unmet medical need in early Alzheimer's disease: an evaluation of beta-amyloid (Aβ) plaque burden as a potential drug development tool. Clin Pharmacol Ther.

[46] Aduhelm (aducanumab-avwa) [prescribing information]. Biogen; Cambridge, MA. 2022.

[47] Leqembi (lecanemab-irmb) [prescribing information]. Eisai; Nutley, NJ. 2023.

[48] Kisunla (donanemab-azbt) [prescribing information]. Eli Lilly; Indianapolis, IN. 2024.

[49] Bateman R.J., Cummings J., Schobel S., Salloway S., Vellas B., Boada M. (2022). Gantenerumab: an anti-amyloid monoclonal antibody with potential disease-modifying effects in early Alzheimer's disease. Alzheimers Res Ther.

[50] Boxer A.L., Sperling R. (2023). Accelerating Alzheimer's therapeutic development: the past and future of clinical trials. Cell.

[51] Biogen Idec presents positive interim results from phase 1B study of investigational Alzheimer’s Disease treatment Aducanumab (BIIB037) at 2015 AD/PD™ conference. 2015 https://investors.biogen.com/news-releases/news-release-details/biogen-idec-presents-positive-interim-results-phase-1b-study. August 13, 2025.

[52] Sevigny J., Chiao P., Bussière T., Weinreb P.H., Williams L., Maier M. (2016). The antibody aducanumab reduces aβ plaques in Alzheimer's disease. Nature.

[53] Biogen presents data from phase 1b study of investigational Alzheimer’s Disease treatment Aducanumab at 2016 Clinical Trials on Alzheimer’s Disease meeting. 2016 https://investors.biogen.com/news-releases/news-release-details/biogen-presents-data-phase-1b-study-investigational-alzheimers. August 15, 2025.

[54] Chen T., O'Gorman J., Castrillo-Viguera C., Rajagovindan R., Curiale G.G., Tian Y. (2024). Results from the long-term extension of PRIME: a randomized Phase 1b trial of aducanumab. Alzheimer's Dement.

[55] Eisai and Biogen announce positive topline results of the final analysis for BAN2401 at 18 months. 2018 https://investors.biogen.com/news-releases/news-release-details/eisai-and-biogen-announce-positive-topline-results-final. August 15, 2025.

[56] Berry D.A., Dhadda S., Kanekiyo M., Li D., Swanson C.J., Irizarry M. (2023). Lecanemab for patients with early Alzheimer disease: bayesian analysis of a phase 2b dose-finding randomized clinical trial. JAMA Netw Open.

[57] Shcherbinin S., Evans C.D., Lu M., Andersen S.W., Pontecorvo M.J., Willis B.A. (2022). Association of amyloid reduction after Donanemab treatment with tau pathology and clinical outcomes: the TRAILBLAZER-ALZ randomized clinical trial. JAMA Neurol.

[58] Lilly's donanemab slowed Alzheimer's disease progression in Phase 2 trial: full data presented at AD/PD™ 2021 and published in NEJM. 2021 https://lilly.mediaroom.com/2021-03-13-Lillys-donanemab-slowed-Alzheimers-disease-progression-in-Phase-2-trial-full-data-presented-at-AD-PD-TM-2021-and-published-in-NEJM. August 15, 2025.

[59] Budd Haeberlein S., Aisen P.S., Barkhof F., Chalkias S., Chen T., Cohen S. (2022). Two randomized phase 3 studies of Aducanumab in early Alzheimer's disease. J Prev Alzheimers Dis.

[60] Cavazzoni P. FDA's decision to approve new treatment for Alzheimer's disease. 2021 https://www.fda.gov/drugs/our-perspective/fdas-decision-approve-new-treatment-alzheimers-disease. September 20, 2024.

[61] Lecanemab confirmatory phase 3 CLARITY AD study met primary endpoint, showing highly statistically significant reduction of clinical decline in large global clinical study of 1795 participants with early Alzheimer’s Disease 2022 https://investors.biogen.com/news-releases/news-release-details/lecanemab-confirmatory-phase-3-clarity-ad-study-met-primary. August 15, 2025.

[62] van Dyck C.H., Swanson C.J., Aisen P., Bateman R.J., Chen C., Gee M. (2023). Lecanemab in early Alzheimer's Disease. N Engl J Med.

[63] Bateman R.J., Smith J., Donohue M.C., Delmar P., Abbas R., Salloway S. (2023). Two phase 3 trials of Gantenerumab in early Alzheimer's disease. N Engl J Med.

[64] [Ad hoc announcement pursuant to Art. 53 LR] Roche provides update on phase III GRADUATE programme evaluating gantenerumab in early Alzheimer’s disease. 2022 https://www.roche.com/investors/updates/inv-update-2022-11-14c. August 15, 2025.

[65] McDade E., Cummings J.L., Dhadda S., Swanson C.J., Reyderman L., Kanekiyo M. (2022). Lecanemab in patients with early Alzheimer's disease: detailed results on biomarker, cognitive, and clinical effects from the randomized and open-label extension of the phase 2 proof-of-concept study. Alzheimers Res Ther.

[66] U.S. Food and Drug Administration. FDA converts novel Alzheimer’s Disease treatment to traditional approval. 2023 https://www.fda.gov/news-events/press-announcements/fda-converts-novel-alzheimers-disease-treatment-traditional-approval. July 23, 2025.

[67] Lilly's Donanemab Significantly Slowed Cognitive and Functional Decline in Phase 3 Study of Early Alzheimer's Disease. 2023 https://lilly.mediaroom.com/2023-05-03-Lillys-Donanemab-Significantly-Slowed-Cognitive-and-Functional-Decline-in-Phase-3-Study-of-Early-Alzheimers-Disease. August 15, 2025.

[68] Sims J.R., Zimmer J.A., Evans C.D., Lu M., Ardayfio P., Sparks J. (2023). Donanemab in early symptomatic Alzheimer disease: the TRAILBLAZER-ALZ 2 randomized clinical trial. Jama.

[69] U.S. Food and Drug Administration. FDA approves treatment for adults with Alzheimer’s disease. 2024 https://www.fda.gov/drugs/news-events-human-drugs/fda-approves-treatment-adults-alzheimers-disease. July 23, 2025.

[70] Prentice R.L. (1989). Surrogate endpoints in clinical trials: definition and operational criteria. Stat Med.

[71] Freedman L.S., Graubard B.I., Schatzkin A. (1992). Statistical validation of intermediate endpoints for chronic diseases. Stat Med.

[72] Elliot M.R. (2023). Surrogate endpoints in clinical trials. Ann Rev Stat Appl.

[73] Joffe M.M., Greene T. (2009). Related causal frameworks for surrogate outcomes. Biometrics.

[74] Chen T., Tian Y., Millen B. (2025). Surrogate Endpoints in drug development: a review of statistical and regulatory perspectives and applications. WIREs Comput Stat.

[75] Buyse M., Molenberghs G., Burzykowski T., Renard D., Geys H. (2000). The validation of surrogate endpoints in meta-analyses of randomized experiments. Biostatistics.

[76] Chen T., Hutchinson R.M., Rubel C., Murphy J., Xie J., Montenigro P., et al. A statistical framework for assessing the relationship between biomarkers and clinical endpoints in Alzheimer's Disease. J Prev alzheimers Dis. 2024. 10.14283/jpad.2024.126. PMC11436399 39350368

[77] Micheel C.M., Ball J.R. (2010). Evaluation of biomarkers and surrogate endpoints in chronic disease.

[78] Klunk W.E., Koeppe R.A., Price J.C., Benzinger T.L., Devous M.D., Jagust W.J. (2015). The Centiloid Project: standardizing quantitative amyloid plaque estimation by PET. Alzheimers Dement.

[79] Chen T., Hutchison R.M., Tian Y., Rubel C., Murphy J., Xie J. (2023). Presented at the 2023 AD/PD conference.

[80] Wang Y. (2023). An insider's perspective on FDA approval of aducanumab. Alzheimers Dement (N Y).

[81] Sargent D.J., Mandrekar S.J. (2013). Statistical issues in the validation of prognostic, predictive, and surrogate biomarkers. Clin Trials.

[82] Mallinckrodt C., Tian Y., Aisen P.S., Barkhof F., Cohen S., Dent G. (2023). Investigating partially discordant results in phase 3 studies of Aducanumab. J Prev Alzheimers Dis.

[83] Nitsch R. (2022). Presented at Clinical Trials on Alzheimer's Disease (CTAD).

[84] Smith J., Donohue M.C., Gruendl E., Grimmer T., Perry R.J., Black S.E. (2023). Presented at the American Academy of Neurology (AAN) Annual Meeting.

[85] Krudys K. FDA Clinical Review. Aduhelm (aducanumab). 2021.

[86] Barkhof F., Klein G., Tonietto M., Bittner T., Ahlers S., Abbas R. (2023). Presented at the American Society of Neuroradiology (ASNR) Annual Meeting.

[87] John O’Gorman J.M., Montenigro Philip, Shardae Showell, Gersham Dent, Carrie Rubel, Matthew Hutchison R., Chen Tianle, Kumar Kandadi Muralidharan, Kate Dawson. (2023). Pooled ENGAGE/EMERGE integrated placebo-controlled period and long-term extension (LTE) topline results: slower clinical progression at week 134 in aducanumab-treated patients that became amyloid PET negative at week 78. Clinical Trials on Alzheimer’s Disease (CTAD).

[88] Joshi A.D., Pontecorvo M.J., Lu M., Skovronsky D.M., Mintun M.A., Devous M.D. (2015). A semiautomated method for quantification of F 18 florbetapir PET images. J Nucl Med.

[89] Bateman R.J., Smith J., Donohue M.C., Delmar P., Abbas R., Salloway S. (2022). Presented at Clinical Trials on Alzheimer's Disease (CTAD).

[90] Bujkiewicz S., Thompson J.R., Riley R.D., Abrams K.R. (2016). Bayesian meta-analytical methods to incorporate multiple surrogate endpoints in drug development process. Stat Med.

[91] Aisen P., Bateman R.J., Crowther D., Cummings J., Dwyer J., Iwatsubo T. (2024). The case for regulatory approval of amyloid-lowering immunotherapies in Alzheimer's disease based on clearcut biomarker evidence. Alzheimers Dement.

[92] Chen E.Y., Haslam A., Prasad V. (2020). FDA acceptance of surrogate end points for cancer drug approval: 1992-2019. JAMA Intern Med.

[93] Dubois B., von Arnim C.A.F., Burnie N., Bozeat S., Cummings J. (2023). Biomarkers in Alzheimer's disease: role in early and differential diagnosis and recognition of atypical variants. Alzheimers Res Ther.

[94] Ackley S.F., Zimmerman S.C., Brenowitz W.D., Tchetgen Tchetgen E.J., Gold A.L., Manly J.J. (2021). Effect of reductions in amyloid levels on cognitive change in randomized trials: instrumental variable meta-analysis. Bmj.

[95] (2022). Effect of reductions in amyloid levels on cognitive change in randomized trials: instrumental variable meta-analysis. Bmj.

[96] Pang M., Zhu L., Gabelle A., Gafson A.R., Platt R.W., Galvin J.E. (2023). Effect of reduction in brain amyloid levels on change in cognitive and functional decline in randomized clinical trials: an instrumental variable meta-analysis. Alzheimers Dement.

[97] Shan G., Ritter A., Miller J., Bernick C. (2022). Effects of dose change on the success of clinical trials. Contemp Clin Trials Commun.

[98] ClinicalTrials.gov. NCT05361382. Head-to-Head Harmonization of Tau Tracers in Alzheimer's Disease (HEAD). https://clinicaltrials.gov/study/NCT05361382. September 20, 2024.

[99] Leuzy A, Raket LL, Villemagne VL, Klein G, Tonietto M, Olafson E, Baker S, Saad ZS, Bullich S, Lopresti B, Bohorquez SS, Boada M, Betthauser TJ, Charil A, Collins EC, Collins JA, Cullen N, Gunn RN, Higuchi M, Hostetler E, Hutchison RM, Iaccarino L, Insel PS, Irizarry MC, Jack CR, Jagust WJ, Johnson KA, Johnson SC, Karten Y, Marquié M, Mathotaarachchi S, Mintun MA, Ossenkoppele R, Pappas I, Petersen RC, Rabinovici GD, Rosa-Neto P, Schwarz CG, Smith R, Stephens AW, Whittington A, Carrillo MC, Pontecorvo MJ, Haeberlein SB, Dunn B, Kolb HC, Sivakumaran S, Rowe CC, Hansson O, Doré V (2024). Harmonizing tau positron emission tomography in Alzheimer’s disease: The CenTauR scale and the joint propagation model. Alzheimers Dement..

[100] Elia E.G., Städler N., Ciani O., Taylor R.S., Bujkiewicz S. (2020). Combining tumour response and progression free survival as surrogate endpoints for overall survival in advanced colorectal cancer. Cancer Epidemiol.

[101] U.S. Food and Drug Administration. Table of surrogate endpoints that were the basis of drug approval or licensure. 2022 https://www.fda.gov/drugs/development-resources/table-surrogate-endpoints-were-basis-drug-approval-or-licensure. September 18, 2024.

[102] Mittal A., Kim M.S., Dunn S., Wright K., Gyawali B. (2024). Frequently asked questions on surrogate endpoints in oncology-opportunities, pitfalls, and the way forward. eClinical Med.

[103] Walia A., Haslam A., Prasad V. (2022). FDA validation of surrogate endpoints in oncology: 2005-2022. J Cancer Policy.

[104] Rafii M.S., Sperling R.A., Donohue M.C., Zhou J., Roberts C., Irizarry M.C. (2023). The AHEAD 3-45 study: design of a prevention trial for Alzheimer's disease. Alzheimers Dement.

