# Supplementary material for: A review of evidence supporting amyloid beta reduction as a surrogate endpoint in Alzheimer’s disease
Source: J Prev Alzheimers Dis. 2026 Jan 1;13(2):100458. doi: 10.1016/j.tjpad.2025.100458 (PMC12869041; doi:10.1016/j.tjpad.2025.100458)
Supplement: Supplementary file 1 [file mmc1.docx]

# **SUPPLEMENTARY MATERIAL**

### **Supplementary Table 1.** Clinical studies used for analyses of Aβ reduction as a surrogate endpoint for slowing of clinical decline in Alzheimer’s disease

| **Drug name** | **Clinical study (phase)** | **Intervention* (administration)** | **Patients randomized, n** | **Amyloid PET population, n** | **Clinical efficacy endpoints^‡^** |
| --- | --- | --- | --- | --- | --- |
| Aducanumab [1-3] | PRIME NCT01677572  (phase 1b) | Fixed dose of 1, 3, 6, and 10 mg/kg and titration to 10 mg/kg (IV Q4W) | 197 | 167 | CDR-SB  MMSE |
|  | EMERGE  NCT02484547  (phase 3) | Low dose: titration to 3 mg/kg for *APOE* ε4 carriers and 6 mg/kg for non-carriers (IV Q4W).  High dose: titration to 10 mg/kg^†^ (IV Q4W) | 1643 | 488 | CDR-SB  MMSE  ADAS-Cog13  ADCS-ADL-MCI |
|  | ENGAGE  NCT02477800  (phase 3) |  | 1653 | 585 |  |
| Lecanemab [4, 5] | BAN2401-G000-201  NCT01767311  (phase 2b) | 2.5 mg/kg (IV Q2W),  5 mg/kg (IV Q4W),  5 mg/kg (IV Q2W),  10 mg/kg (IV Q4W),  and 10 mg/kg (IV Q2W) | 854 | 315 | ADCOMS  ADAS-Cog14  CDR-SB  MMSE |
|  | CLARITY AD  NCT03887455  (phase 3) | 10 mg/kg (IV Q2W) | 1795 | 698 | CDR-SB  ADAS-Cog14  ADCOMS  ADCS-ADL-MCI |
| Gantenerumab [6] | GRADUATE I  NCT03444870  (phase 3) | Titration to 510 mg  (SQ Q2W) | 985 | 123 | CDR-SB  ADAS-Cog13  ADCS-ADL  FAQ  MMSE |
|  | GRADUATE II  NCT03443973  (phase 3) |  | 980 | 114 |  |
| Donanemab [7, 8] | TRAILBLAZER-ALZ ^¶^  NCT03367403  (phase 2) | 3 doses of 700 mg and 1400 mg thereafter  (IV Q4W) | 257 | 233 | iADRS  CDR-SB  ADAS-Cog13  ADCS-iADL  MMSE |
|  | TRAILBLAZER-ALZ 2  NCT04437511  (phase 3) | 3 doses of 700 mg and 1400 mg thereafter  (IV Q4W) | 1736^§^ | 1577 |  |

* Only active treatment groups are listed; each study had a placebo control group. ^†^ Was 6 mg/kg for *APOE* ε4 carriers before protocol version 4. **^‡^** In the order of the study design. ^¶^ Patients with an SUVR >1.46 were considered to have a high tau level and were excluded; patients with a SUVR <1.10 or with a deposition pattern inconsistent with AD were considered to have an inadequate tau level and were excluded. Patients with an SUVR <1.10 but with a topographic deposition pattern consistent with advanced AD were included [7]. ^§^ Patients with low/medium or high tau pathology. Abbreviations: Aβ, amyloid beta; ADAS-Cog, Alzheimer’s Disease Assessment Scale–Cognitive Subscale; ADCOMS, Alzheimer’s Disease Composite Score; ADCS-ADL, Alzheimer’s Disease Cooperative Study–Activities of Daily Living; ADCS-ADL-MCI, Alzheimer’s Disease Cooperative Study–Activities of Daily Living Inventory for Mild Cognitive Impairment; ADCS-iADL, Alzheimer’s Disease Cooperative Study–Instrumental Activities of Daily Living; *APOE*, apolipoprotein E; CDR-SB, Clinical Dementia Rating–Sum of Boxes; FAQ, Functional Activities Questionnaire; iADRS, Integrated Alzheimer’s Disease Rating Scale; IV, intravenous; MMSE, Mini Mental State Examination; PET, positron emission tomography; SQ, subcutaneous; SUVR, standardized uptake value ratio; Q2W, every 2 weeks; Q4W, every 4 weeks.

### **Supplementary Table 2. Individual-level correlation between change from baseline in amyloid PET and clinical endpoints in EMERGE at Week 78**

|  |  |  | **Partial Spearman correlation^a^** | |
| --- | --- | --- | --- | --- |
| **Aβ endpoint** | **Clinical endpoint** | **Hypothesized correlation direction** | **Placebo** | **Active treatment arm** |
| Amyloid PET composite SUVR | CDR-SB | Positive | −0.06 | 0.19** |
|  | MMSE | Negative | 0.23 | −0.24*** |
|  | ADAS-Cog13 | Positive | −0.17 | 0.20** |
|  | ADCS-ADL-MCI | Negative | 0.11 | −0.29*** |

Correlation between Aβ reduction and clinical efficacy endpoints from baseline at Week 78.
*P* values (nominal): ** *P*<.01 and *** *P*<.001.

^a^Correlations are partial Spearman correlations assessed after adjustment for baseline biomarker and efficacy values. The active treatment arm analyses were based on the pooled low- and high-dose groups.

Abbreviations: Aβ, amyloid β; ADAS-Cog13, Alzheimer’s Disease Assessment Scale–Cognitive Subscale (13 items); ADCS-ADL-MCI, Alzheimer’s Disease Cooperative Study–Activities of Daily Living Inventory for Mild Cognitive Impairment; CDR-SB, Clinical Dementia Rating–Sum of Boxes; MMSE, Mini Mental State Examination; PET, positron emission tomography; SUVR, standardized uptake value ratio.

**References:**

[1] Budd Haeberlein S, Aisen PS, Barkhof F, Chalkias S, Chen T, Cohen S, et al. Two Randomized Phase 3 Studies of Aducanumab in Early Alzheimer's Disease. J Prev Alzheimers Dis. 2022;9:197-210. 10.14283/jpad.2022.30.

[2] Sevigny J, Chiao P, Bussière T, Weinreb PH, Williams L, Maier M, et al. The antibody aducanumab reduces Aβ plaques in Alzheimer's disease. Nature. 2016;537:50-6. 10.1038/nature19323.

[3] Chen T, O'Gorman J, Castrillo-Viguera C, Rajagovindan R, Curiale GG, Tian Y, et al. Results from the long-term extension of PRIME: A randomized Phase 1b trial of aducanumab. Alzheimer's & Dementia. 2024;20:3406-15. <https://doi.org/10.1002/alz.13755>.

[4] Berry DA, Dhadda S, Kanekiyo M, Li D, Swanson CJ, Irizarry M, et al. Lecanemab for Patients With Early Alzheimer Disease: Bayesian Analysis of a Phase 2b Dose-Finding Randomized Clinical Trial. JAMA Netw Open. 2023;6:e237230. 10.1001/jamanetworkopen.2023.7230.

[5] van Dyck CH, Swanson CJ, Aisen P, Bateman RJ, Chen C, Gee M, et al. Lecanemab in Early Alzheimer's Disease. N Engl J Med. 2023;388:9-21. 10.1056/NEJMoa2212948.

[6] Bateman RJ, Smith J, Donohue MC, Delmar P, Abbas R, Salloway S, et al. Two Phase 3 Trials of Gantenerumab in Early Alzheimer's Disease. N Engl J Med. 2023;389:1862-76. 10.1056/NEJMoa2304430.

[7] Mintun MA, Lo AC, Duggan Evans C, Wessels AM, Ardayfio PA, Andersen SW, et al. Donanemab in Early Alzheimer's Disease. N Engl J Med. 2021;384:1691-704. 10.1056/NEJMoa2100708.

[8] Sims JR, Zimmer JA, Evans CD, Lu M, Ardayfio P, Sparks J, et al. Donanemab in Early Symptomatic Alzheimer Disease: The TRAILBLAZER-ALZ 2 Randomized Clinical Trial. Jama. 2023;330:512-27. 10.1001/jama.2023.13239.
